# Immediate psychological effects of COVID-2019 in people sheltered in place living in New York state

**DOI:** 10.1192/j.eurpsy.2021.1784

**Published:** 2021-08-13

**Authors:** I. Piretti, M. Benassi, F. Ambrosini, R. Sant’Angelo

**Affiliations:** 1 Psychologist, Hr Specialist, Independent Researcher, Cesena, Italy; 2 Department Of Psychology, University of Bologna, Cesena, Italy; 3 Psychologist, Independent Researcher, Savignano, Italy; 4 Mental Health Department, AUSL ROMAGNA, Cesena, Italy

**Keywords:** shelter in place, Anxiety, stress, Insomnia

## Abstract

**Introduction:**

The epidemic caused by the SARS-CoV-2, which began in Wuhan city in December 2019, quickly spread to various countries around the world. The first case in New York State was confirmed on March 1; three weeks later (on March 22, 8 p.m.) the entire population was sheltered in place (SIP). By March 27, the USA had already become the first country in the world for the number of infections. 56% of known domestic cases were confined to New York State.

**Objectives:**

The study aims to evaluate the immediate psychological effects on sheltered in place persons aged between 18 and 70 years old and living in New York State (USA).

**Methods:**

This study is based on a cross-sectional online survey conducted anonymously in the period between the tenth and twenty-third day of SIP. Zung Anxiety Self-Assessment Scale (ZAS scale), Insomnia Severity Index (ISI) and Perceived Stress Scale 4 (PSS4) were used to evaluate anxiety, insomnia and stress respectively.

**Results:**

We collected data on 354 individuals (189 females, 34.9 years). MANOVA evidenced that anxiety was significantly related to marital status (higher for divorced/widow participants as compared to married/civil partnership and single), it decreased significantly with age, it was higher for females and for persons having an history of psychiatric disorders and sleeping problems.

**Conclusions:**

Our results could be used as a “psychological baseline” meanwhile the outbreak of COVID-19 is still ongoing. Despite the few days of shelter in place, we found the presence of a significant incidence and pervasive prevalence of psychological distress.

**Disclosure:**

No significant relationships.

